# Some Peculiar Eye Symptoms

**Published:** 1885-04

**Authors:** 


### SOME PECULIAR EYE SYMPTOMS.

Sensation as if eyes were being forced out: Bell., Berb., Carbo-v., Card.-b., Caust., Ign., Laur., Led., Lyc., Mag.-c., Ph-ac., Ran.-b., Senega, Thuja.
—as if eyes were pressed into head: Bell., Calc., Caust., Daph.-ind., Kali-c., Zinc.
—as if eyes were fallen in: China.
—as if eyes projected: Bell., Guai.
—as if eyes were coming out: Acon.
—as if cold water between eyelids: Berb.
—as if eye moved involuntarily: Calc.
—as if something moved in the eye, relieved by rubbing: Carb.-an.
—as if a skin were drawn over the eyes: Caust., Ol.-an.
—as if the eyes were swimming in water: China (right and at night), Sil. (l.)
—as if eyes were smaller: Crocus.

